# Longitudinal changes of clavarioid funga (Basidiomycota) diversity in the tundra zone of Eurasia

**DOI:** 10.1080/21501203.2017.1345801

**Published:** 2017-07-03

**Authors:** Anton G. Shiryaev

**Affiliations:** Institute of Plant and Animal Ecology, Russian Academy of Sciences, Ekaterinburg, Russia

**Keywords:** Arctic, biogeography, climate change, distribution, spatial turnover, permafrost

## Abstract

The study deals with certain variations of the diversity level of clavarioid funga in the 33 localities (100 km^2^ each) inside seven longitudinal sectors (100,000 km^2^) situated along the gradient of the climatic continentality of the Eurasian tundra zone. As continentality increases, from the maritime climate of Fennoscandia to the continental climate of Yakutia, α-diversity and γ-diversity decrease considerably. On the other side, spatial turnover of species, or β-diversity, grows in the direction of continental areas. This paper uses the following methods to assess the spatial turnover: Whittaker’s index and mean Jaccard similarity index, as well as by several other parameters. In addition, our data show that the genus *Typhula* is richest in the tundra, and its share in the structure of the clavarioid funga grows as the studied area decreases, as well as when the environmental conditions become more severe (continentality of the climate). Also, the paper discusses the issue of newly emerging taiga fungi species in the European Arctic, which is connected with climatic warming followed by ″greening″ of the tundra. Note that in the cryo-semiarid continental climate of Yakutia, where climatic changes are just as pronounced, no new taxons have been discovered so far.

## Introduction

The Arctic is one of the world’s four regions that were classified by the intergovernmental group of climatic change experts as territories with the most obvious changes (Katsov and Porfiriev 2012). The growth rate of temperature in the Eurasian part of the tundra zone is 0.43ºС/10 years, and this growth rate will be maintained in the near future (IPCC 2014).

″Greening″ of many sectors of the Arctic has been noted, and it is reflected in significant changes of the biodiversity level of the ecosystems situated in the cryolithozone (Shiyatov and Mazepa 2007; Forbes et al. 2010). The biota is restructured, which is indicated by ″forest″ boreal species that were not present in the tundra zone before or those that can be found solely in the intro-zonal bottomland biotopes. Thanks to current climatic changes, these species appear in water sheds and oust aggressively conventional cryophilic elements of the tundra biota. At the same time, increasing diversity of the flora in the European tundra as a whole and those specific local floras are more obvious here compared to continental regions of Siberia (Yurtsev et al. 2004; Walker et al. 2012; Belonovskaya et al. 2016). Traditionally, these processes are studied on the basis of various groups of animals and plants, whereas representatives of the Fungi Kingdom are extremely uncommon to be used as model groups (Dahlberg et al. 2013; Geml et al. 2016).

In this study, clavarioid fungi (Basidiomycota) are suggested as a model group – the group whose spatial distribution has been relatively well studied in the Northern Eurasia (Shiryaev 2014). Clavarioids are distributed on all continents, from the polar deserts and alpine glaciers to the tropical deserts and equatorial rain forests. This group has about 670 described species (IndexFungorum 2017). They are macromycetes, characterised by bright, visible basidiomes, reaching 20 kg and 0.6 m in diameter (species of genus *Sparassis*), which can be preserved for a long time in herbaria. Clavarioids combine three major functional groups of fungi: parasites, saprotrophs, symbionts (incl. mycorrhizal and ″basidiolichens″), thereby participating in the key processes of the biosphere (soil formation and nutrient cycling), and are basic species of primary succession, as well as plant pathology.

Species richness of the clavarioid fungi in Eurasia probably is higher in the hemiboreal zone (269 species) with optimal hydro-thermal conditions and decline to the tundra (46 species). Only six species are found in the native conditions of the arctic deserts with pessimal conditions (lack of thermoresources) (Shiryaev 2013a, 2014, 2015a). In this case, the clavarioid fungi of the tundra zone are poor compared with the boreal or nemoral variants.

Clavarioid funga (mycobiota, mycota, etc.) of the High Arctic is very specific. There are species of two genera only: *Typhula* and *Multiclavula* (Shiryaev 2014, 2015a). Moreover, as regards the native conditions of the Arctic desert, the above funga is the only one capable of growing fruit bodies, not only among clavarioids, but also among all Aphyllophorales, that is, this funga is best of all adapted among Aphyllophorales to the thermal criophilic pessimum on the planet (Shiryaev 2015a).

It has been shown recently that, on the global and continental scales, the taxonomical and ecologo-morphological structure of clavarioids varies considerably not only from the South to the North, but also from the West to the East, that is, as the climate becomes more continental, from the coast of the Atlantic and Pacific Oceans to the centre of the continent. Within the forest zone of the northern Eurasia, for the regional fungas (each of them – 100,000 km^2^ on mean), the trend of linear decreasing has been established for the species richness of the clavarioid fungi – on mean, two times, compared to the maritime regions, in the direction of ultra-continental Yakutia and Mongolia (Shiryaev 2014, 2015b).

For the clavarioid funga of the tundra zone, a similar trend was found during studies of seven longitudinal sectors situated to the North off the Polar Circle, from Fennoscandia to Chukotka. Currently, the total number of clavarioid species known in the tundra zone of Fennoscandia, which the sector has the optimum bioclimatic conditions for development of the clavarioids, accounts for 38 species. For the other side, only 26 species have been discovered in the harsh cryo-semiarid continental conditions of Yakutia. Thus, γ-diversity decreases by one-third as continentality increases (Shiryaev 2013a, 2014). Recently, similar results were obtained for the Arctic deserts (Shiryaev 2015a).

The accumulated data enable us to study the longitudinal gradient based on a smaller scale – in ″localities″ (100 km^2^ each). Will the diversity of the clavarioid funga maintain the decreasing trend as continentality increases if the territory under study is reduced by three orders of magnitude? Will there be any changes of α-diversity and β-diversity (or species turnover)? Traditionally, ultra-continental regions are characterised by the maximum heterogeneity level of the bioclimatic conditions (Gaston 2000; Lomolino et al. 2010); how will these specifics affect the clavarioid funga? Which taxonomical groups prevail in different scales? These are the questions we will attempt to answer in this study. This paper can be regarded as the ″starting point″, after which it will be possible to monitor changes in the tundra’s funga according to the current global climate changes, particularly reflected in the high-latitude ecosystems.

The basic aim of this study is estimating the clavarioid funga diversity based on an assessment of species richness in the localities distributed through the seven longitudinal sectors of the tundra zone of Eurasia with increasing climatic continentality (from the mild maritime climate of Fennoscandia to the harsh ultra-continental climate in Yakutia). In addition, the following issues will be discussed: is there an increase in the heterogeneity of funga diversity (species turnover) with increasing heterogeneity of the environment to the ultra-continental climate? Is there any relation between different levels of diversity with basic bioclimatic factors? Which species are the most widespread in the tundra zone, and which of them appear in some restricted sectors only?

## Materials and methods

### Site description

Despite the fact that most of the Eurasian tundra zone is situated along sea coasts, there are Fennoscandia and Chukotka sectors which are located in the area that is directly impacted by the Atlantic and Pacific Oceans, which determine milder conditions compared to Siberian sectors distinguished by pronounced continental features (Rivas-Martinez et al. 2011). The Eurasian tundra zone (by the example of zone Е, Walker et al. 2005) is subdivided into seven longitudinal sectors (each of them – 100,000 km^2^ on mean). For instance, continentality index of Fennoscandia is 23, whereas it increases twice in continental Yakutia, up to 48 (Table 1). The continentality index is an integral indicator, along which, apart from the difference between the mean annual temperatures of the coldest and warmest months, many other bioclimatic indicators vary distinctly, too. For example, these include decreasing of the mean annual temperature, of the maximum and minimum temperature, of the sum of active temperatures (Σ*t*>10°С), of the mean annual volume of precipitation and of the days without frost. On the other hand, as the climate becomes more continental, the area of territories with permafrost increases (from 15% to 100%) and so does the thickness of the permafrost (from less than 50 m to more than half of kilometre) (Tumel 2002). There is no doubt that changes of these parameters determine considerably the diversity of biota, including the clavarioid fungi.

**Table 1.** Bioclimatic parameters for the seven longitudinal sectors of Eurasian tundra.

Parameter	Longitudinal sector						
	Fenno-scandia	Kanin-Pechora	The Urals	Yamal-Gydan	Taimyr	Yakutia	Chukotka
Mean annual temperature, °С ± SD	+1.5 ± 1.1	−3.5 ± 1.3	−5.1 ± 1.4	−6.8 ± 1.4	−12.6 ± 1.7	−13.5 ± 1.9	−8.5 ± 1.8
Mean annual temperature of coldest month, °С	−10.5	−17.3	−21.7	−24.6	−32.5	−37.3	−25.2
Mean absolute minimum temperature, °С	−40.5	−47.6	−50.4	−56.1	−59.2	−57.3	−49.9
Mean Σ*t*>10°С	515	430	390	350	260	220	375
Mean annual precipitation, mm ± SD	523 ± 23	456 ± 22	551 ± 30	430 ± 21	280 ± 26	217 ± 29	336 ± 30
Mean number of frost-free days	106	75	61	58	45	34	55
Permafrost territory, %	15	40	75	91	100	100	99
Permafrost thickness, m	<50	<50	100	100–300	300–500	>500	100–300
Mean index continentality	23	30	35	40	44	48	40

The second scale used in this study is the locality (100 km^2^). There are 33 localities studied inside the seven longitudinal sectors: six localities are in the Atlantic (Fennoscandia) and Pacific Ocean sector (Chukotka), and five in the over sectors (Kanin-Pechora, the Urals, Yamal-Gydan, Taimyr, Yakutia) (Supplement materials, Table 1). The choice of localities was based on the following criteria: absence of recent fires and strong anthropogenic impact; the distances from anthropogenic sites (e.g. highways, railways, garbage storage) should exceed at least 100 m; native habitats should be old-growth, or should have been free from any anthropogenic disturbance for a long time.

### Sampling

The author has been studying the clavarioid funga of the Eurasian tundra for the past 20 years already. Part of the materials was collected during the International Transsiberian Mycological Expedition. Specimens collected by the author are deposited in the herbarium (mycological department of SVER) of the Institute of Plant and Animal Ecology of the Russian Academy of Sciences (Ekaterinburg).

The specimens were collected in the localities during at least two seasons, but most of them have been studied by different researchers for three and more years. The parameter of 90% of species richness from the best studied locality is used as a unit of measurement to estimate the lowest level for the number of collection records needed from each locality. For example, there are 25 species presented by 440 records (specimens, notes in the diary, photos, etc.) in Liinahamari – the best studied locality of Fennoscandia (Russia, Murmansk province, Pechenga area, 69º39ʹ N, 31º22ʹ E). In this case, 90% of 440 records are 390 records. All localities used for analysis in this sector include 390 records and more. This parameter applies to the sector situated in the mild climate of the Eurasian tundra because of the warming impact of the Gulfstream. For the harsh ultra-continental climate of Yakutia the best studied locality Chersky (Nizhnekolymsk area, 69°02ʹ N, 160°43ʹ E) includes 19 species. There are 450 records and more in the localities in Yakutia included in the current study.

The paper discusses the species found solely within 33 localities, distributed between seven longitudinal sectors. The list does not include the species that are listed in the literature for the Arctic if their taxonomical positions are currently uncertain: *Clavulinopsis arctica* Kobayasi, *Clavulina amethystenoides* (Peck) Corner, *Pistillaria diaphana* Fr., *P. pusilla* Fr., *Typhula gyrans* Fr. Also from this work deleted the species found only at the human-modified habitats *T. trifolii* Rostr., *Artomyces pyxidatus* (Pers.) Jülich, *Ramaria stricta* (Pers.) Quél. So, the base of the paper is currently valid list of species in order to find the correlation between the diversity and current bioclimatic factors and their variations.

There are publications dedicated to certain longitudinal sectors and localities used in the work: (1) Fennoscandia (Shiryaev 2009, 2013a, 2013b, 2014; Shiryaev and Mukhin 2010; Knudsen and Vesterholt 2012); (2) Kanin-Pechora (Shiryaev 2012a, 2014); (3) the Urals (Kazantseva 1970; Shiryaev 2006a, 2006b, 2010, 2013a, 2014, 2015a); (4) Yamal-Gydan (Shiryaev 2008, 2010, 2013a, 2014); (5) Taimyr (Shiryaev 2010, 2011, 2013a, 2014); (6) Yakutia(Shiryaev 2012b; Shiryaev and Mikhaleova 2013); (7) Chukotka (Shiryaev 2013c, 2014). Recently, a paper was published, which was dedicated to diversity and distribution of the Aphyllophoroid fungi, including clavarioid life form, in the Arctic desert zone (Shiryaev 2015a). The method of localities (or similar) is widely used for studies of spatial structure of large areas in botany (Malyshev 1994; Ricklefs et al. 2004; Yurtsev et al. 2004) and mycology (Mukhin 1993).

### Data analyses

Several taxonomic parameters were estimated, for example, total number of species in each longitudinal sector (γ-diversity); number of species within a locality; the mean number of species per locality (α-diversity). The degree of changes in species composition along environmental gradients, or spatial turnover of species (β-diversity), was estimated as a ratio between the total and mean species richness (Whittaker’s index). As an alternative, estimation of species turnover was used following the well-known parameter, such as the mean level for Jaccard similarity index (*J*) measured as similarity between all pairs of localities inside the same sector. The low level of similarity reflects high species turnover.

As a surrogate for β-diversity estimating, the mean standard deviation (SD), the coefficient of variation (CV) as the ratio of standard deviation to mean number of species in a sector ((SD/*M*)×100%) and the difference between species richness of the richest and poorest localities (*D*_RPL_, %) are also used. The high levels of these parameters reflect the high levels of species turnover.

Results were obtained using the software STATISTICA 8.0 (StatSoft 2008). The dendrogram is constructed using the Ward’s method and Euclidian distance. A non-parametric Mann–Whitney U-test was used to detect significant differences between the numbers of species in localities distributed by different longitudinal sectors. There is the Spearman’s rank correlation coefficient (*r*_s_) measured for study of correlation between species richness and climatic parameters. Rarefaction curves for observed fungi species richness were calculated using EstimateS Win 9.10 (Colwell and Elsensohn 2014). In the text, the term ″species richness″ means a number of species.

The CLAVARIA^WORLD^ database was created by the author and has been regularly updated over the last 20 years. This resource contains information from over 445 different geographical units worldwide and aggregates currently about 77,000 records that can be found in various check-lists, online databases and herbarium records.

## Result and discussion

### Longitudinal changes of species richness in localities

Forty two species of the clavarioid fungi have been found in 33 localities situated in the tundra zone (Supplement materials, Table 2) that account for 94% of the total number of species currently known in the native conditions of this natural zone of Eurasia (Shiryaev 2014 as amended). Aggregation of these data within the boundaries of the respective seven continentality sectors enables us to state that the maximum number of species has been found in the sectors (γ-diversity) situated near the ″oceanic″ coasts: in Fennoscandia (37 species) and in Chukotka (36) (Table 2). As the climate becomes more continental, γ-diversity decreases down to 26 species in the Siberian sectors. In general, γ-diversity found for each sector includes over 90% of the total number of species known in these sectors. In some sectors (e.g. Yakutia), this value is 100%.

**Table 2.** Clavarioid funga diversity in the seven longitudinal sectors of Eurasian tundra.

Parameter	Longitudinal sector						
	Fenno-scandia	Kanin-Pechora	The Urals	Yamal-Gydan	Taimyr	Yakutia	Chukotka
γ-diversity	37	30	33	26	26	26	36
Maximum	26	24	25	21	20	19	22
Minimum	22	18	18	16	14	11	17
α-diversity	24.2	21.4	21.4	18.7	16.7	14.6	19.8
Whittaker’s index	1.53	1.40	1.54	1.39	1.55	1.78	1.87
SD of species richness	1.48	2.41	2.70	2.06	2.50	3.36	1.92
CV, %	6.1	11.3	12.6	11.0	15.0	23.0	9.7
*D*_RPL_, %	15	25	38	24	30	42	23
Mean *J* of species richness	0.55	0.60	0.57	0.54	0.42	0.40	0.43
Continentality index	23	30	35	40	44	48	40
Number of localities	5	5	5	4	4	5	5

CV – coefficient of variation, %; *D*_RPL_ – difference between richest and poorest localities, %; *J* – Jaccard similarity index.

As regards localities, the maximum and minimum numbers of species vary just as considerably as in the sectors. Both parameters decrease steadily as the climate becomes more severe (continental). For instance, 26–22 species in Fennoscandia decrease down to 19–11 species in Yakutia. Therefore, the mean number of species in the localities (α-diversity) decreases from 24.2 in Fennoscandia to 14.6 in Yakutia (Table 2). In this study, the distribution of species richness is characterised by normal distribution, or very similar. In this case, it is possible to use parametric statistics (mean parameters, SD, CV, etc.). Nevertheless, a small amount of data is analysed, for which purpose it is recommended to use the non-parametric statistics, for example, the median and Mann–Whitney U-test.

**Figure 1.**
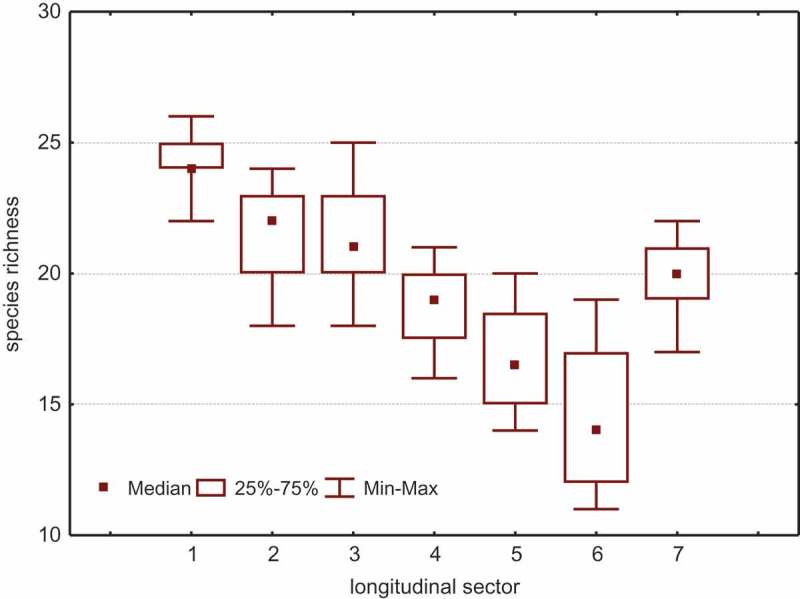
Species richness median of clavarioid funga in localities distributed by seven longitudinal sectors in Eurasian tundra zone. Sector: 1 – Fennoscandia, 2 – Kanin-Pechora, 3 – the Urals, 4 – Yamal-Gydan, 5 – Taimyr, 6 – Yakutia, 7 – Chukotka.

The variation trend of the species richness median is very close to the mean species richness (α-diversity) and changing from Fennoscandia to Chukotka (from 24.1 to 15.0 species, respectively) (Figure 1).

No statistically valid difference has been established between α-diversity levels in the adjacent sectors (*p* > 0.05, Mann–Whitney U-test); but, the decreasing trend is obvious as the climate becomes more continental (*r*_s_ = −1.00, *p* < 0.01). Nevertheless, there is a difference between the mean number of species in the European and Siberian sectors (Figure 1). The number of species in the European sectors varies from 26 to 18, and in the Siberian sectors – from 21 to 11. In the European and Siberian sectors, α-diversity (22.3 and 16.5) and the median (22.8 and 16.9) are different with a high probability level (*р* < 0.01); for comparison, the Student *t*-criterion and the Mann–Whitney U-test are used. The Chukotka localities, including from 22 to 17 species, do not differ from the European and Siberian ones in terms of the α-diversity (19.8) and the median (20.0) (*р* > 0.05).

**Figure 2.**
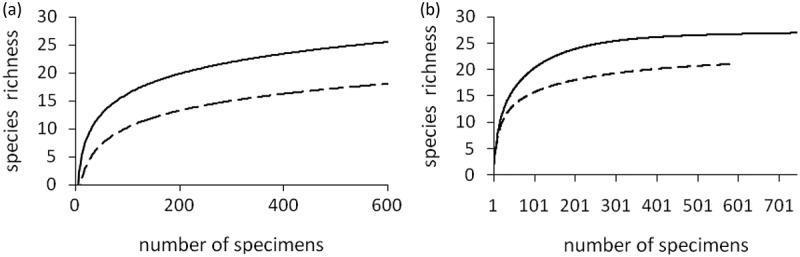
(a) Rarefaction curves for the best observed localities in Fennoscandia and Yakutia, (b) Jackknife 1 estimator for predicted number of species. Line – Fennoscandia (Liinahamari, Murmansk province); dotted line – Yakutia (Chersky).

The study involves certain localities with the well-identified species composition of the fungi. Accumulation curves of the species richness for the identified records are shown for the two best-studied localities that present the sectors with ″opposite″ richness levels: Fennoscandia (locality Liinahamari) and Yakutia (locality Chersky). Despite the high number of species collected (25 and 19), the species accumulation curves have not reached the asymptote, though most of them have declining slopes and are approaching their asymptotes (Figure 2(a)). According to the estimator Jackknife 1 (Figure 2(b)), these numbers represent 92.6% and 90.5% of the predicted richness (27 and 21 species). Similar results were observed for each locality, which is sufficient for comparing species composition among the areas.

### Longitudinal changes of spatial turnover

As shown previously, as the climate becomes more continental, α-diversity and γ-diversity decrease (Table 3). However, decreasing in the mesoscale (α-diversity) is more pronounced when compared to the macroscale (γ-diversity) (1.67 and 1.42, respectively), so that β-diversity estimated as a ratio between γ- and α-diversity (Whittaker’s index) increases together with continentality: from 1.40 in one of European sector to 1.87 in Chukotka (Table 2), that is, it grows by one-third. Therefore, β-diversity grows in the reverse direction compared to changes of α- and γ-diversities.

**Table 3.** Correlation of clavarioid funga diversity and bioclimatic parameters in the Eurasian tundra zone.

Parameter	γ-diversity	α-diversity	Whittaker’s index	Mean *J* of species richness	SD of species richness	CV, %
Continentality index	−0.89*	−1.00**	0.43 ^n.s.^	−0.86*	0.61 ^n.s.^	0.77*^.^
Mean annual temperature	0.75*	0.96**	−0.64 ^n.s.^	0.89*	−0.57 ^n.s.^	−0.68 ^n.s.^
SD of mean annual temperature	**−0.54**^n.s.^	−0.82*	0.82*	−0.79*	0.57 ^n.s.^	0.61 ^n.s.^
Absolute minimum temperature	0.86*	0.93**	−0.29 ^n.s.^	0.71 ^n.s.^	−0.76*	−0.79*
Mean Σ*t*>10°С	0.86*	1.00**	−0.43 ^n.s.^	0.86*	−0.61 ^n.s.^	−0.75*
Mean number of frost-free days	0.75*	0.96**	−0.64 ^n.s.^	0.89*	−0.57 ^n.s.^	−0.68 ^n.s.^
Mean annual precipitation	0.64 ^n.s.^	0.86*	−0.54 ^n.s.^	0.89*	−0.32 ^n.s.^	−0.5 ^n.s.^
SD of mean annual precipitation	0.14 ^n.s.^	−0.14 ^n.s.^	0.79*	−0.21 ^n.s.^	0.39 ^n.s.^	0.29 ^n.s.^
Permafrost territory	−0.75*	−0.93**	0.61 ^n.s.^	−0.86*	0.5 ^n.s.^	0.64 ^n.s.^

* – *p *< 0.05; ** – *p *< 0.01; ^n.s.^ – not significant, *p *> 0.05. Correlation measured as a Spearman’s coefficient (*r*_s_). Significant results are in **bold**.

In general, the differentiating diversity (β-diversity) can be assessed by various methods. For example, as the climate becomes more continental in the tundra zone, the SD increase 2.3 times (from 1.48 to 3.36) and the CV grows 3.8 times (from 6.1% to 23.0%). It is interesting to note how the difference changes between the poorest and the richest localities (*D*_RPL_) within each sector: in Fennoscandia, the poorest locality had 22 species, and the richest one – 26, that is, the difference is 15%, while in Yakutia, this parameter increases almost three times, up to 42% (11 and 19 species, respectively). As the climate becomes more continental, the mean level of Jaccard’s similarity coefficient decreases from 0.60 to 0.40, and some localities in Yakutia situated relatively close feature the similarity coefficient as low as 0.29, which is the evidence of the high level of environmental heterogeneity determined by the maximum continentality level of the climate.

The increase of β-diversity is indirectly proven by the assessment of the constant in the Michaelis–Menten equation. For instance, it is necessary to collect 81 specimens in the Fennoscandian localities to find half of the species richness, whereas 143 specimens have to be collected in Yakutia; it means that β-diversity is 1.8 times higher in Yakutia (Figure 2(а)). Also, we may mention here that, in order to identify in Yakutia the same number of fungi species as in Fennoscandia (13.5 species accounting for 50% of the potential species number in the locality), three times more sampling efforts are required. In other words, environmental heterogeneity in the continental conditions has a stronger impact on the diversity of fungas. This conclusion confirms the results obtained for fungas of the ″southern″ natural zones, even though the tundra ecosystems are traditionally considered to be arranged in a simple manner.

**Figure 3.**
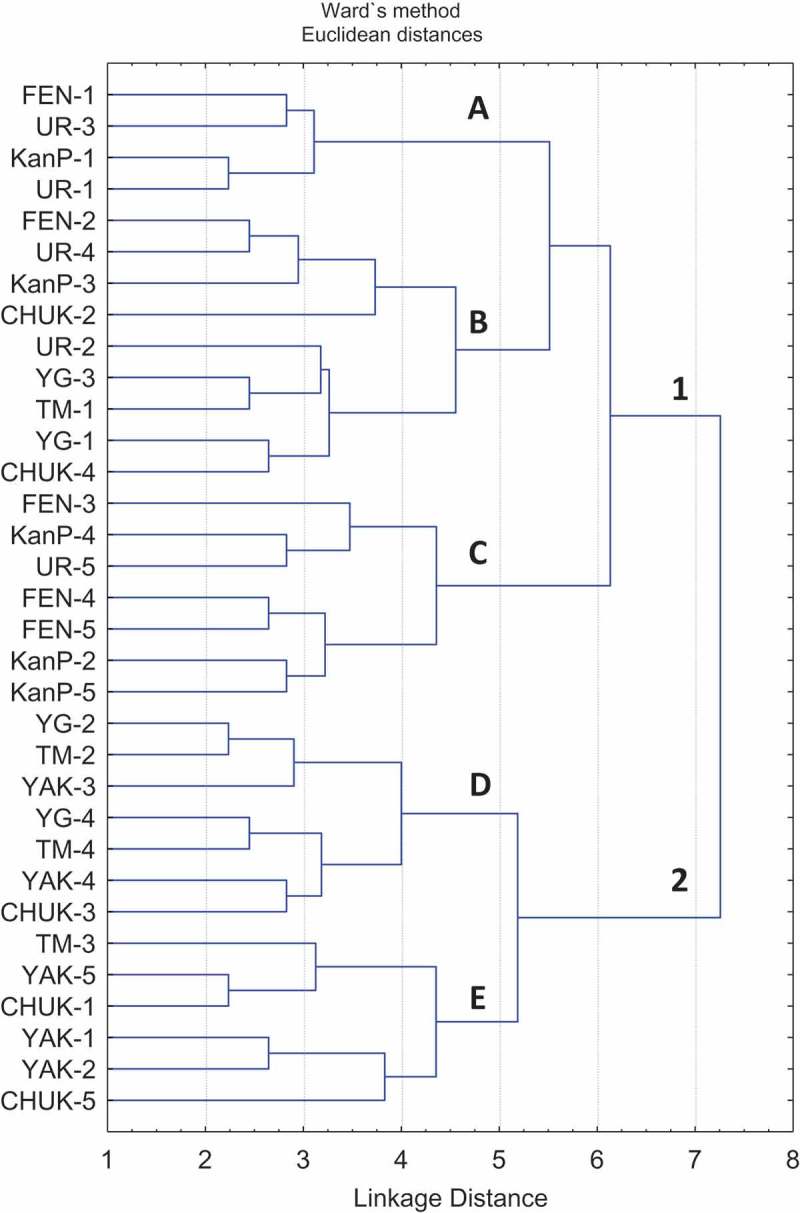
Dendrogram of **s**imilarity between 33 studied localities in the Eurasian tundra. Abbreviation of localities according Supplement materials, Table 1.

Environmental heterogeneity is also reflected in the distribution of localities in a dendrogram based on the similarity of the species richness levels and the set of species. It has been established in the sectoral scale that the maritime sectors are rich, and the continental ones are poor. The species richness analysis of localities (Supplement materials, Table 2) proves this conclusion (*p* < 0.01). The dendrogram including 33 localities divides them into two clusters – rich localities in the optimum conditions (cluster 1) and poor ones in pessimal conditions (cluster 2), that is, just as on the sectoral level, there is the split into ″sub-ocean″ and ″continental″ conditions (Figure 3). As the scale of the localities is studied, it appears that certain continental localities have a similar level of the species richness and a set of species with relation to their ″sub-ocean″ counterparts. Such localities (YG-1, YG-3, TM-1) are mostly situated near large rivers (Ob, Yenisei), which create milder bioclimatic conditions promoting the local development of a more complex funga. Even in continental regions with pessimal climate and edaphic conditions, some ″fragments″, refugiums of the optimum conditions remain. In other words, the relatively complex localities are joined by two localities from the Yamal-Gydan sector and one from Taimyr. There are no localities from continental Yakutia in cluster 1, and there are no localities from the ″optimum conditions″ in the ″pessimal cluster″ (2). Group А includes the richest localities including the great number of ″western″ sub-oceanic species. Here we see solely localities of Fennoscandia, Kanin-Pechora and the Ural. Completely opposite of it is Group Е including the localities situated in the most pessimal continental conditions (Taimyr, Yakutia and western Chukotka), some of the poorest localities including the maximum number of ubiquist species.

This distribution of the clavarioid fungi along the Eurasian tundra zone is yet another evidence of the similarity of the species richness level and the structure of the high-latitude biota of Fennoscandia and the Ural (these facts have been mentioned more than once during flora analysis (Dahl 1963; Hämet-Ahti 1981; Humphrise et al. 1999) as well as various groups of cryptogrammic organisms: lichens and mosses (Ahti 1977; Urbanavichus 2011; Mateo et al. 2016).

### Relationship between clavarioid funga diversity and bioclimate

Сontinentality index has a negative significant correlation (Table 3) with γ-diversity (*r*_s_ = −0.89, *р < *0.05), whereas the relation is considerably higher for α-diversity (*r*_s_ = −1.00, *р < *0.01). As regards the parameters describing the β-diversity, the significant correlation is set forth for the mean Jaccard similarity coefficient and CV, whereas the Whittaker’s index and the SD are below the significance level.

Studies of relations between the diversity and the climatic parameters show that richness peaks of γ-diversity of the clavarioid funga correspond with Fennoscandia and Chukotka (Table 2); however, the mean annual temperature and precipitation in these sectors are considerably different (Table 1). Both these parameters are considerably higher on the Atlantic coast (Mann–Whitney U-test, *p* > 0.05), while the Chukotka sector is statistically similar to the Siberian one (*p* < 0.01). As regards the γ-diversity level, significant dependency has only been found for the mean annual temperature (*r*_s_ = 0.75, *p* < 0.05), whereas it is not found for the mean annual precipitation (*r*_s_ = 0.64, *p* > 0.05) (Table 3). This result is the indirect evidence of the fact that the biota diversity in the Eurasian tundra zone is determined predominantly by the temperature factor compared to the humidity level. This conclusion confirms the overall global principle regarding the prevailing role of the lack of thermal resources in the planet’s high latitude for the biota diversity (Lomolino et al. 2010). As regards γ-diversity, the tipping point on the longitudinal gradient corresponds to the boundary between the Ural and Yamal-Gydan at the mean annual temperature from −5.5ºС to 6.5ºС (Table 1), that is, the funga under consideration is divided into the European and Asian parts. The significant correlation for β-diversity has been found for the mean Jaccard’s similarity coefficient only.

As the area under study decreases to the level of localities (Figure 3), it appears that α-diversity of the fungi correlates with both the main climatic factors: with the mean annual temperature (*r*_s_ = 0.96, *p* < 0.01) and precipitation (*r*_s_ = 0.86, *p* < 0.01).

The important differentiation parameter of funga diversity is the level of active temperatures (the sum of temperatures above 10ºС). It has a linear significant correlation with α-diversity (*r*_s_* *= 1.00, *p* < 0.01), and slightly less so with γ-diversity (*r*_s_* *= 0.86, *р* < 0.05). As regards β-diversity, significant correlation is found for the Jaccard’s coefficient and the CV.

α-diversity depends significantly on the mean number of frost-free days (*r*_s_* *= 0.96, *p* < 0.01), while this climatic parameter is less significant for γ-diversity (*r*_s_* *= 0.75, *p* < 0.05). As regards β-diversity, significant dependency is found for the mean Jaccard’s similarity coefficient (*r*_s_* *= 0.89, *p* < 0.05).

The critical factor forming the funga diversity in the tundra zone is the presence of permafrost. α- and γ-diversities are found to have a significant negative correlation with the increasing spreading of permafrost. A similar result has been received for the Jaccard’s coefficient, too, whereas this parameter shows no statistical confidence for Whittaker’s index and the variation coefficient.

Studies of the aforementioned examples show that one can hardly speak about similar significance of the same bioclimatic factors for funga formation of sectors and localities (at different scales of studies). It is obvious that different diversity levels react in a different way to the bioclimatic factors at different scales. Probably, in order to resolve this issue, apart from the modern ecological factors discussed herein, of importance is also the historical factor that determines the long development period of the biota without permanent glaciations within Beringia, whereas the Last Glacial Period expired in Fennoscandia 9600 years ago only.

There are no special studies of diversity for over morphological and taxonomic groups of Aphyllophoids along the gradient of continentality in the tundra zone. Nevertheless, our own practice and several publications demonstrate that cantharelloid and stipitate hydnoid fungi disappear, and the species richness of poroid fungi declines (Kotiranta and Mukhin 2000; Mukhin and Kotiranta 2001; Shiryaev and Mikhaleva 2013). There are similar trends that were found in the boreal-forest zone for the poroid and cantharelloid fungi (Parmasto 1977; Petrenko 1978; Mikhaleva 1993; Mukhin and Ushakova 2003), as well as for sclerotioid phytopatogenic fungi causing the ″snow mold″ (Hoshino et al. 2010).

### Changes of taxonomic parameters

As shown in different studies, the genus *Typhula* is richest in the tundra zone of Eurasia; it accounts for about 55% of total species richness of the clavarioid fungi (e.g. Shiryaev 2013a, 2014). In this work, the genus *Typhula* has 57% (24 species) of the total list (Supplement materials, Table 2).

On the sectoral level, shares of *Typhula* are not statistically different between adjacent continentality sectors (*р* > 0.05); however, there is an obvious growth trend of the share of the *Typhula* from the Atlantic coast to the Pacific Ocean (from 54% to 64%), that is, *Typhula* in all cases account for more than half of all the clavarioid species.

Studies of the species composition in localities show that the average share of *Typhula* in sectors varies from 58% to 77%, growing from Fennoscandia in the eastern direction. There are some Yakutian and Chukotka localities, where the share of *Typhula* is as high as 82%: for instance, the species of genus *Typhula* account for 9 out of 11 species found in the poorest localities under study (№ 24) (Supplement materials, Table 2). Therefore, as the area of the territory under study decreases, the share of *Typhula* grows (*р* < 0.05). If we consider continental localities only, confidence of this conclusion increases (*р* < 0.01). This result is also indirectly confirmed by the fact that a similar conclusion has been reached for clavarioid fungi existing in the forest-tundra zone of Eurasia (Shiryaev 2014). In general, it is established that high share of the genus *Typhula* reflects externality of the bioclimatic conditions in high latitudes and ultra-continental territories (Shiryaev 2014, 2015a, 2015b).

Various fungal species are characterised by different occurrence in the tundra zone. For example, certain species have been found in more than three-fourth localities (number of localities/% of 33): *T. lutescens* (33/100), *T. setipes* (31/94), *T. caricina* and *T. crassipes* (30/91), *T. variabilis* (28/85), *T. culmigena* (27/82), *Clavaria argillacea* (26/79), *C. falcata* and *Pterula gracilis* (25/76), *T. erythropus, T. sclerotioides* and *T. uncialis* (24/73). All these species are typical ubiquists in the northern Eurasia. Most of them are saprotrophs on the decaying grasses, herbs and leaves. On the other hand, the following species were found in one to three localities (3–9%) only: *Clavulina rugosa, Clavulinopsis corniculata, Clavulinopsis laeticolor, Macrotyphula fistulosa, Ramaria abietina, T. curvispora, T. ishikariensis, T. muelleri, T. pertenius, T. sphaeroidea, T. umbrina*. Such species have their ecological optimum in the boreal and nemoral forests. In the tundra zone they are found in the ″oceanic″ sectors only. Because the Gulfstream creates a mild climate, there is a small square of the Fennoscandian tundra there with permafrost and annual temperatures below zero. In this case, several typical ″forest″ species could grow here on the soil and litter, e.g., *Clavulina rugosa, Clavulinopsis corniculata, Clavulinopsis laeticolor, R. abietina*, but they are not present in the continental areas.

Over the last 5 years, new clavarioid species typical predominantly of the ″forest″ ecosystems have been discovered more and more often in the high-latitude regions of the European part of the Arctic (Shiryaev 2015b); this can be explained by warming of the climate and by expansion of the forest vegetation into previously forestless ″tundra″ regions (Walker et al. 2012). On the other hand, no new species have been registered in continental regions of Yakutia during the same period. Of interest is the fact that the region is totally dominated by ubiquists and that there are no ″typically forest″ boreal species, as well as no north-bound expansion of areas is observed for any boreal species already discovered. This fact is quite noteworthy. Climate researches have established that Yakutia is one of the Siberian regions where the mean annual temperature has risen most considerably over the last 50 years; however, there have been almost no changes of the vegetation structure and of permafrost thickness (Gavrilova 2005; IPCC 2014). The biota of the north-eastern Asia remains unchanged and maintains the structure that was formed in the ″Ice Age″. The area is totally dominated by rare suffruticose plants alternating with tundra herbs; however, larch forests and associated bushes (as representatives of the taiga biota) that grow solely along river bodies (e.g. at the mouth of the Lena River on Tit-Ary Island, 72º N) never appear in watersheds. It may so happen that as the warming continues, development of the biota in this region will lag behind considerably from the potentially possible level (Herzschuh et al. 2016). Our results indirectly confirm these thoughts.

## Conclusion

Traditionally, diversity distribution is studied on latitude and longitude gradients, whereas studies of continentality, the third gradient, are extremely uncommon. Our studies prove that α- and γ-diversities of the boreal clavarioid funga decrease as continentality increases in the moderate climate of Eurasia. This paper shows that it also takes place within tundra zone. From the ″sub-oceanic″ sectors (Fennoscandia and Chukotka) towards continental regions of Yakutia, γ-diversity decreases 1.42 times, and α-diversity decreases 1.66 times.

A typical feature of the ultra-continental climate is the increased heterogeneity of the bioclimatic conditions manifested by the great difference between annual, seasonal and daily rhythms, which affects development rhythms of plants and heterotrophs, including various groups of funga. Heterogeneity of the ultra-continental regions also affects the structure of the clavarioid funga resulting in growing β-diversity (as opposed to α- and γ-diversities). For example, in Yakutia, within the tundra zone, following parameters reach the maximum possible level: CV; the ratio between the total and mean numbers and of species in a locality (Whittaker’s index); the difference between species richness of the richest and the poorest localities and the SD. As continentality increases, the mean level of Jaccard’s similarity coefficient decreases. Increasing heterogeneity of funga under study is also reflected by the fact that in Yakutia, one has to take three times more sampling efforts to find a similar number of species, compared to Fennoscandia.

These results demonstrate that the clavarioid funga in the tundra zone are poor (compared to the forest fungas), but, contrary to the widespread belief, they are not so simple and unified. Of interest is the fact that the tundra funga demonstrates significant differences in the diversity levels between the ″sub-oceanic″ coasts of the Atlantic and the Pacific Oceans compared to continental territories of Taimyr and Yakutia. Note that significant diversity differences have also been found between Fennoscandia and Chukotka, which is likely to prove the importance of the historical factor for any subsequent studies of the respective fungas.

These studies have just begun, and obviously, they have to involve more localities. In future, such studies would help create a single biogeographic map of northern Eurasia. Currently, all biogeographic maps of the world are based solely on the laws governing the distribution of animals and plants, while the representatives of the Kingdom Fungi have not been included into these constructions.
